# High-resolution small RNA structures from exact nuclear Overhauser enhancement measurements without additional restraints

**DOI:** 10.1038/s42003-018-0067-x

**Published:** 2018-06-07

**Authors:** Parker J. Nichols, Morkos A. Henen, Alexandra Born, Dean Strotz, Peter Güntert, Beat Vögeli

**Affiliations:** 1 Department of Biochemistry and Molecular Genetics, University of Colorado Denver, Anschutz Medical Campus, 12801 East 17th Avenue, Aurora,, CO 80045 USA; 2 Faculty of Pharmacy, Mansoura University, Mansoura, 35516 Egypt; 3 Laboratory of Physical Chemistry, ETH Zürich, ETH-Hönggerberg, Zürich, 8093 Switzerland; 4 Institute of Biophysical Chemistry, Center for Biomolecular Magnetic Resonance, Goethe University Frankfurt am Main, Frankfurt am Main, 60438 Germany; 5 Graduate School of Science, Tokyo Metropolitan University, Hachioji, Tokyo 192-0397 Japan

## Abstract

RNA not only translates the genetic code into proteins, but also carries out important cellular functions. Understanding such functions requires knowledge of the structure and dynamics at atomic resolution. Almost half of the published RNA structures have been solved by nuclear magnetic resonance (NMR). However, as a result of severe resonance overlap and low proton density, high-resolution RNA structures are rarely obtained from nuclear Overhauser enhancement (NOE) data alone. Instead, additional semi-empirical restraints and labor-intensive techniques are required for structural averages, while there are only a few experimentally derived ensembles representing dynamics. Here we show that our exact NOE (eNOE) based structure determination protocol is able to define a 14-mer UUCG tetraloop structure at high resolution without other restraints. Additionally, we use eNOEs to calculate a two-state structure, which samples its conformational space. The protocol may open an avenue to obtain high-resolution structures of small RNA of unprecedented accuracy with moderate experimental efforts.

## Abstract (web summary)

Parker Nichols et al. present an exact nuclear Overhauser enhancement (eNOE) protocol for defining small RNA structures at high resolution using only NOE distance data. They apply eNOE to a 14-mer UUCG tetraloop structure, obtaining a decrease in root-mean-square deviation from 1.52 Å to 0.44 Å, compared to conventional NOE.

## Introduction

Although Nuclear Magnetic Resonance (NMR)-determined structures currently make up <10% of the total in the Protein Data Bank (PDB), they account for >40% of all RNA structures. This is mostly due to the inherent advantages of NMR over other structural techniques when it comes to RNA. First, solution-state NMR completely avoids the common difficulties involving crystallization of RNA common in X-ray^1, 2^. Second, NMR is one of the most powerful techniques for studying interactions between proteins, other nucleic acids, low molecular weight molecules, and solvent molecules. Finally, NMR is well-equipped to probe the inherent dynamics of RNA molecules, proven critical to their functions by amassing evidence^3–5^. In particular, residual dipolar couplings (RDCs) have been shown especially fruitful for investigating such dynamics, as they are capable of reporting on the orientation of bond vectors relative to a known molecular alignment frame^6^. Although X-ray crystallography and cryo-electron microscopy (cryo-EM) are still the techniques of choice for investigating RNAs and RNA–protein complexes larger than 50 kDa, which are difficult to study using NMR due to spectral overlap and fast *T*_2_ relaxation times, recent methodological advances have made NMR an alternative for studies of RNA of such sizes^7–11^.

Despite these advantages, NMR has room for substantial improvement. One such area is the continued use of distance-dependent Nuclear Overhauser Enhancements (NOE) rate constants as semiquantitative upper limit distance restraints^12^, which are employed this way due to various interfering mechanisms throughout the pulse sequence^13^, but mainly spin diffusion and dynamics^14^. The non-exact nature of these restraints means that important information about structure and dynamics is lost. Therefore, the only current NMR methods for probing dynamics are spin relaxation measurements, which are usually used for probing single-site flexibility and exchange, and RDCs, which require high technical sophistication when applied to RNA^15^.

RNA poses several challenges over proteins regarding NOE spectroscopy (NOESY). As a biopolymer composed of only four chemically unique building blocks, as opposed to 20 for proteins, RNA results in a large amount of spectral overlap, causing resonance assignment to be more difficult. This is exemplified by the H2′-H5′′ ribose protons whose chemical shifts normally appear within the narrow range of 4–5 ppm where water signal predominates. The overlap problem is further increased by the predominately A-form helical structure of RNA, which results in a lack of chemical environment diversity, especially for larger RNAs. Chemical shift diversity is therefore often only seen in non-canonical RNA structures such as hairpin loops, bulges, or internal loops^16^. An additional difficulty with using NOEs in RNA structure determination is the low proton density of RNA compared with proteins, resulting in a sparser NOE network. The lack of sufficient NOE distance restraints means that traditional structure calculations have to rely on additional restraints such as dihedral angle restraints, RDCs, cross-correlated relaxation (CCR) rates^17–19^, electron paramagnetic resonance measurements^20^, as well as hydrogen bonding patterns, and rarely can these RNA structures achieve high resolution with NOEs alone^16, 21^. Often, semi-empirical restraints such as base-pair planarity are added that cannot be used to characterize spatial sampling because they lack an accurate parameterization relating a specific conformation in a dynamic ensemble to an empirical observable. Therefore, a better use of the NOE would improve the quality of NMR structures and enable spatial sampling to be probed.

**Fig. 1:**
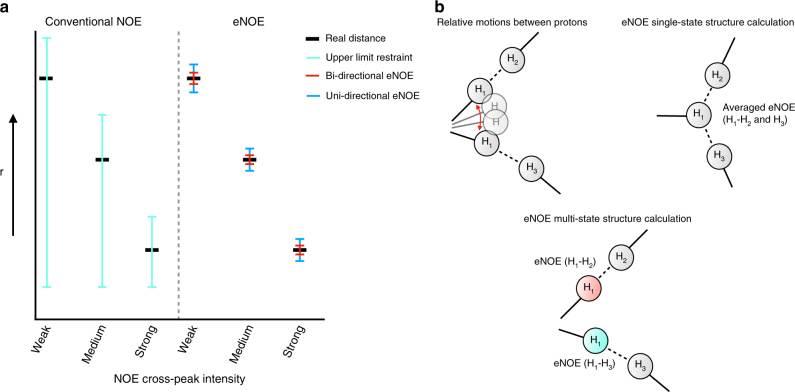
The eNOE principle. **a** Although conventionally measured NOEs are converted into upper limit distance restraint with a relatively loose tolerance, eNOE-derived restraints have an upper and lower limit. If both NOESY cross peaks caused by a spin pair can be evaluated, the tolerance is very tight (red). If only one cross-peak can be used, the tolerance is somewhat less stringent, in the present application ± 10%. **b** The eNOE is a time-averaged quantity. For a mobile atom, H_1_ sampling positions both close to protons H_2_ and H_3_, NOEs H_1_–H_2_ and H_1_–H_3_ suggest proximity of H_1_ to both H_2_ and H_3_. The combined NOE data can be explained better by a model that allows two states for H_1_ shown in red and cyan than by a single-state structure

We have previously reported on the methodology and use of exact nuclear Overhauser enhancements (eNOEs) for the determination of distances up to 5 Å with less than a 0.1 Å error in proteins (Fig. 1a)^22, 23^. The *r*^−6^-averaged nature of eNOEs allows for the construction of multi-state ensembles that describe their conformational space (Fig. 1b)^24, 25^. Although we have applied our eNOE protocol to a number of proteins^25^, we have yet to investigate its applicability to RNA. Interestingly, pioneering work on the extraction of exact distances in biomacromolecules from NOE buildup measurements was carried out on RNA^26^ as early as the late 1980s, most notably by the groups of James^27, 28^, Jardetzky^29^, Kaptein^30^ and Gorenstein^31, 32^. A typical application was the distinction between A, B and D helical forms.

**Fig. 2:**
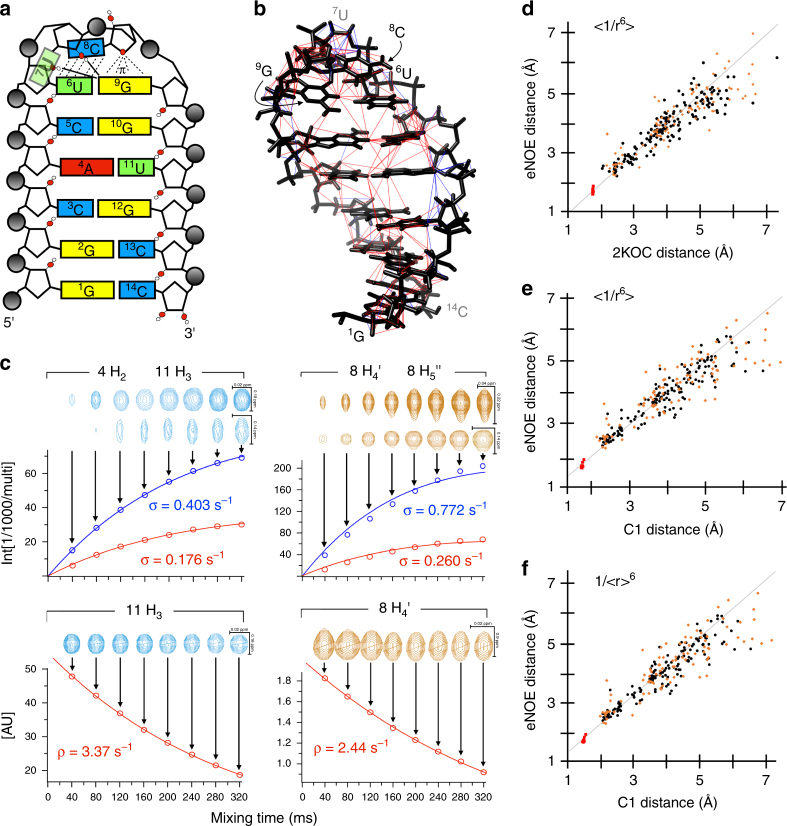
eNOE network, decay and buildup curves and distances. **a** Secondary structure and sequence of the 14-mer UUCG tetraloop and notable defining features. Hydrogen bonds between the hydroxyls of U6/U7 and the base of G9 are indicated by solid black lines. The base-stacking interaction between U8 and U6 and the O_4’_-π stacking contacts between the C8 ribose and the G9 purine are shown by dashed black lines. **b** The 265 extracted eNOEs (red) and 88 gn-eNOEs (blue) plotted onto the tetraloop structure 2KOC^14^. **c** Bi-directional *σ*_*ij*_ buildup curves from fitting the cross-peak intensities from 40 to 20 ms mixing time between 4H_2_ and 11H_3_ in the H_2_O NOESY spectrum are shown in the top left, and the corresponding fit to the 11H_3_ diagonal peak intensities is shown below. A similar case is shown on the right side for the 8H_4’_ and 8H_5′′_ atoms of the D_2_O NOESY spectrum. The peaks from which the intensities were extracted are shown above the fits. For the bi-directional fits, the top peaks correspond to *σ*_*ij*_ (blue) and the bottom peaks to σ_*ji*_ (green). **d**‒**f** Correlation plots between distances from 2KOC and C1 on the *x* axis and eNOE distances from fits of 40 to 160 ms on the *y* axis. Black circles correspond to distances between non-amino/non-methylene protons, orange diamonds to distances between amino/methylene protons and non-amino/methylene or amino/methylene protons on a different residue, and red squares to distances within amino/methylene spin pairs. The 2KOC distances **e** were determined by taking *r*^−6^ averages ( < 1/r^6^ > ) (overall correlation: *y* = 0.96 *x* , *R* = 0.89; black circles: 0.96 x , 0.89; orange diamonds: 0.96 x , 0.84; red squares: 0.98 x , 0.35). The C1 distances **e** were determined by taking *r*^−6^ averages (overall: 0.97 x , 0.89; black circles: 0.98 x , 0.90; orange diamonds: 0.96 x , 0.80; red squares: 0.98 x , 0.60). The C1 distances **f** were linearly averaged distances (<r>) (overall: 0.95 x , 0.89; black circles: 0.96 x , 0.91; orange diamonds: 0.94 x , 0.82; red squares: 0.98 x , 0.59)

To determine the feasibility and accuracy of extracting eNOE distances from RNA, we have applied our eNOE protocol to the RNA 14-mer UUCG tetraloop^16, 21, 33–37^. The aptly named four-nucleotide tetraloops are simple but important RNA structural motifs that stabilize the caps of RNA stem loops^38^ and have other functions including initiation of RNA folding^39, 40^, participation in tertiary interactions in large RNAs such as ribosomal RNAs^41–43^ and self-splicing RNAs^44^, and as recognition sites for proteins in ribonucleoprotein complexes^45–47^. The secondary structure of the 14-mer, as well as the discussed defining features of the UUCG tetraloop are shown (Fig. 2a**)**. The high thermodynamic stability of the UUCG tetraloop has been attributed to arise from a non-canonical base-pair between U6 and G7 of the loop, favorable base-stacking between U6 and U8, and stabilizing hydrogen bonds between the U6 and U7 hydroxyls and the purine of G9^48^. In addition, the UUCG tetraloop adopts a Z-turn motif that is defined by favorable O_4’_-π stacking contacts between the ribose of C8 and the purine of G9^49, 50^. Here, we present evidence that eNOEs do not only provide interproton distances to high accuracy, but also contain enough information to define RNA structures to high resolution with no additional restraints.

## Results

### Accuracy of extracted eNOE distances

To reliably determine the NOE cross-relaxation rate constants for our unlabeled 14-mer UUCG tetraloop (see Methods), we excluded overlapped diagonal and cross peaks from analysis. This substantially reduced the number of distance restraints because of the large amount of spectral overlap in the 2D NOESY spectra. We could fit 265 eNOEs out of the totally available 677 semiquantitative NOEs. The NOESY acquired from the sample in H_2_O yielded 91 eNOEs involving exchangeable amino resonances, as well as the stable hydroxyl resonance of U6. The Watergate suppression of the water signal in the H_2_O NOESY was sub-par, therefore, a NOESY with water presaturation was acquired in D_2_O, which yielded 174 eNOEs between non-exchangeable base and ribose resonances. The eNOE coverage of the ribose sugars was poor due to their extreme overlap of the H_2’_ through H_5′′_ resonances in the 2D NOESY. However, this problem was alleviated by the use of 88 generic normalized eNOEs (gn-eNOEs)^51^, which are used as an upper distance limit, as described in Methods. The large number of eight mixing times allowed us to establish a general rule for the optimal NOESY mixing times for RNA (see details in the Methods section). Assuming an inverse relationship of the maximal mixing time with the overall tumbling time *τ*_*c*_, we obtain a maximal mixing time of 4 × 10^−10^ s^2^ *τ*_*c*_^−1^. This is larger than our recommendation of 2.5 × 10^−10^ s^2^ *τ*_c_^−1^ for proteins^26^. The eNOE network spans most of the 14-mer (Fig. 2b). One notable exception was G1, whose amino and imino protons were not present in the NOESY spectrum due to chemical exchange, likely from end-fraying. Additionally, the non-exchangeable resonances of G1 were overlapped due to a lack of chemical shift dispersion. Excluding clear outliers and fits from peaks with low signal-to-noise, the fits were of high quality. Examples of some exemplary cross-peak buildup and diagonal peak decay curves are shown (Fig. 2c). These results indicated that for the 14-mer tetraloop enough eNOEs of good quality could be extracted to ensure the structure calculation.

To investigate the accuracy of the extracted eNOEs distances, we compared the eNOE distances from fits of 40–160 ms (a detailed analysis for this choice of mixing times is presented in the Methods section; Supplementary Data 1) to the previously solved NMR structure with Protein Data Bank accession code 2KOC with an overall root-mean-square deviation (RMSD) of 0.37 Å^21^. 2KOC is a well-defined structure with input restraints from a large set of conventional NOEs, allowed dihedral angle ranges, and RDCs, making it an ideal reference structure. Effective distances were calculated from the 20-conformer 2KOC ensemble by taking the *r*^−6^ average ( < 1/*r*^6^ > ), which takes into account structural variation. This analysis showed that the determined eNOE distances correlate well with the back-calculated distances from 2KOC (Fig. 2d**)**. Despite the good correlation, there were quite a few outliers suggesting that the distance-averaged nature of the eNOEs is sensitive enough to pick up dynamics within the 14-mer. Because 2KOC is an averaged representation of its input data, we also compared our eNOE data set with the 10-state bundle provided by Al-Hashimi (hereafter referred to as C1), which was calculated using RDCs from multiple alignment conditions and is thus more likely to represent the dynamic nature of the 14-mer^15^. However, in order to generate different alignment tensors, the stem of the tetraloop had to be extended and modulated through base mutations in a bulge between what would normally be the stem and UUCG tetraloop of the 14-mer^15^. To still enable a comparison, we discarded all atoms between the gUUCGc loop and the four stem base pairs in all 10 states and all distances between the stem and the loop. Again, the correlation between our eNOEs and the averaged distances was good (Fig. 2e). Interestingly, the correlations to both 2KOC and C1 were essentially of the same quality (2KOC: *R* = 0.89, C1: *R* = 0.89), suggesting that the measured eNOE data set was in good agreement with both structures. We also compared our eNOE data set to the linearly averaged distances < *r* > from C1 (Fig. 2f**)**. The correlation was of the same quality, however, there were several distances that agreed much better with the *r*^−6^ averaging than the averaged distance, indicative of the eNOEs sensitivity to motional effects. Overall, it is clear that the extracted eNOE distances are consistent with the previously determined high-resolution structures.

### eNOEs improve calculated structures

**Fig. 3:**
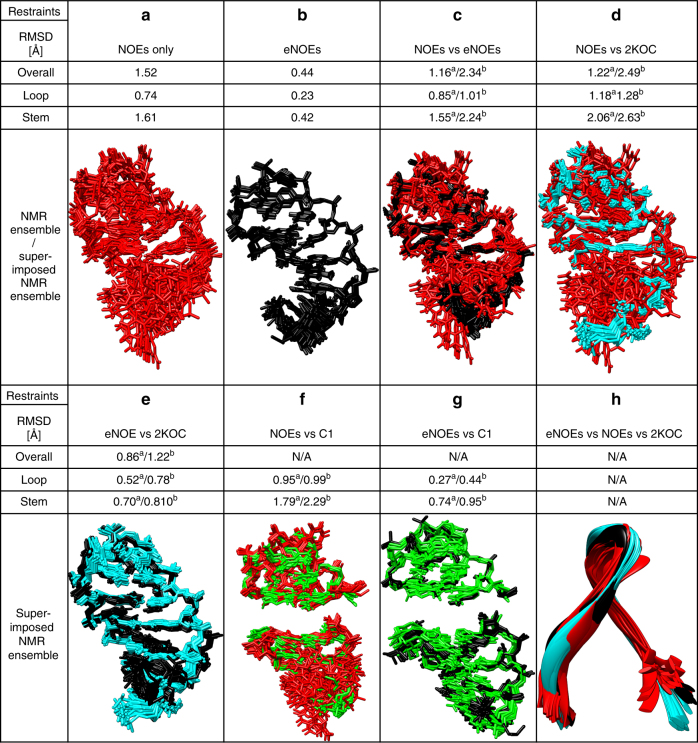
eNOEs improve structures. **a** Structure calculated with only conventional NOEs as input data. **b** Structure calculated using only eNOEs as input data. For **a** and **b** the RMSD values are given for the heavy atoms of nucleotides 1–14 (overall), 1–5 and 10–14 (stem), or 6–9 (loop). Both structures were calculated in CYANA. **c** Superimposition of the structures calculated from conventional NOEs only (red) and eNOEs (black). The RMSD values refer to the deviation between the eNOE and NOE structures. **d** Superimposition of the conventional NOE structure (black) and 2KOC (cyan). **e** Superimposition of the eNOE structure (black) and 2KOC (cyan). **f** Superimposition of conventional NOE structure (black) and C1 (green). The overall RMSD is not available because the loop and stem region had to be evaluated separately. **g** Superimposition of eNOE structure (black) and C1 (green). The overall RMSD is not available because the loop and stem region had to be evaluated separately. **h** Ribbon representations of the NOE structure (red), eNOE structure (black), and 2KOC (cyan). For **c**–**h**, bundle-averaged structures were created using MOLMOL^69^. The RMSD values for the superimposed structures were determined in Chimera^70^ using MatchMaker. For comparisons of structures, the first RMSD (‘^a^’) is the one between the averaged bundles, and the second RMSD (‘^b^’) the one between the bundle of the first reported structure and the averaged second structure

RNA structures calculated from only conventional NOE upper distance bounds are often under-defined due to the low density of NOE restraints. This means that structure calculations are normally supplemented with additional restraints such as Watson–Crick base pairs, dihedral angle ranges, and RDCs, which are determined from other NMR experiments. We have previously shown for the protein GB3 that eNOEs alone contained as much information as traditional NOEs combined with abundant RDC and *J*-coupling data^52, 53^. Therefore, we set out to investigate to what extent our eNOEs could define the 14-mer UUCG tetraloop by themselves. To do this, we calculated single-state structures from the 677 conventional NOEs, or the 353 eNOEs (75 bi-directional, 190 uni-directional, and 88 gn-eNOEs) alone, with no additional restraints such as, for example, base pairing and sugar pucker restraints. The increase in precision when using eNOEs as opposed to conventional NOEs is impressive, which results in an RMSD decrease from 1.52 Å for the conventional NOE structure (Fig. 3a) to 0.44 Å for the eNOE structure (Fig. 3b). One striking observation is the degree of agreement of the conventional NOE structure and the eNOE structure with 2KOC. Although the conventional NOE structure has an overall, loop, and stem RMSD of 1.22, 1.18, and 2.06 Å when compared with 2KOC (Fig. 3d), the eNOE structure has corresponding values of 0.86, 0.52, and 0.70 Å (Fig. 3e). The eNOE structure also agrees much better with the 2KOC structure than the conventional NOE structure in the loop and stem regions (Figs. 3c, e), indicating that the eNOEs alone have a similar information content as the NOEs, RDCs, dihedral angle ranges, Watson–Crick base pairing, and planarity restraints that had been used as input for the 2KOC structure. The poorest agreement between the eNOE and 2KOC structures was observed for the last base-pair between G1 and C14, which lacks eNOE restraints as mentioned before. We also compared the loop and stem regions of our conventional NOE and eNOE structures to the C1 ensemble (Fig. 3f, g). The two structures agree better with C1 than with 2KOC, presumably because C1 is expected to sample a more realistic conformational space.

We also calculated α, β, γ, δ, ε, ζ, χ, η_2_, ν_1_, and ν_2_ (note that δ, ν_1_, and ν_2_ are rigidly linked to each other) dihedral angles from both the conventional NOE and eNOE structures, and 2KOC ensembles (Supplementary Fig. 1 and in the correlation plot in Supplementary Fig. 2). This analysis shows that most of the dihedral angles calculated from the eNOE structure either agree with the 2KOC structure or are very close to it. Although conventional NOEs were able to determine some of the dihedral angles with similar accuracy to those from eNOEs, their precision is not nearly as good (compare error bars in Supplementary Fig. 2). The largest deviations from 2KOC are observed for α, η_2_, and γ. The deviation for the α and η_2_ dihedral angles were expected because they both define angles from which we were not able to measure NOEs, α because it mostly defines the phosphate moiety, and η_2_ because most of the hydroxyls were absent in the spectrum due to chemical exchange at 25 °C. U6 contained the only visible hydroxyl due to its stabilizing role in the tetraloop, and thus its dihedral angles agree well with 2KOC. Thus, the accuracy and precision of eNOEs were able to define the 14-mer UUCG tetraloop to excellent agreement with previous structures with no additional restraints.

### Two-state eNOE structure and dynamics

**Fig. 4:**
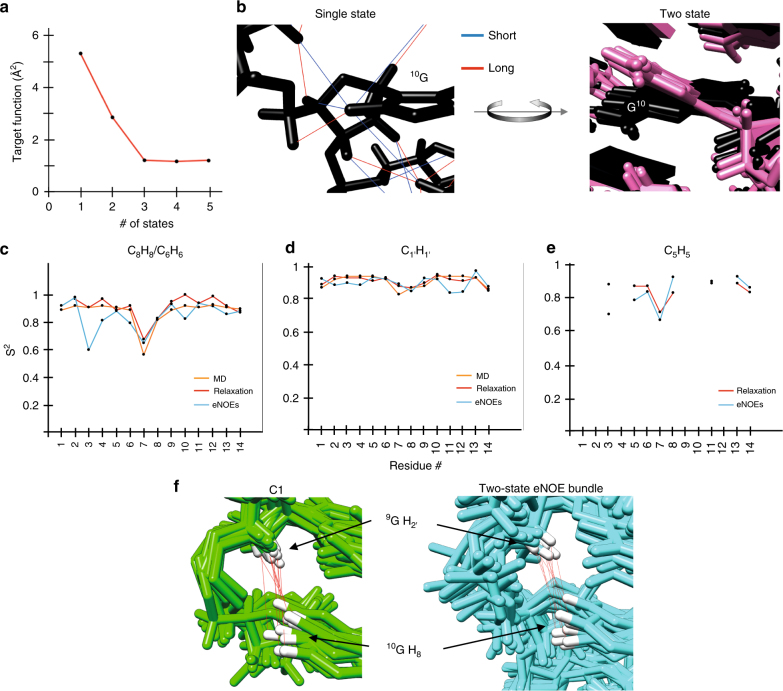
Two-state eNOE structure and sensitivity of eNOEs to motional effects. **a** The number of allowed states in the structure calculation is shown on the *x* axis, and the corresponding CYANA target function on the *y* axis. **b** State separation enforced by distance violation in the single-state structure for G10. Distances that are at least 0.2 Å too short or too long are shown in blue and red, respectively, and the resulting two-state ensemble is represented with five conformers for each state. **c**‒**e** Comparison of order parameters *S*^2^ back-calculated from the two-state ensemble and experimentally and computationally derived *S*^2^. eNOE *S*^2^ of C_8_H_8_ (**c**), C_1’_H_1’_ (**d**) and C_5_H_5_ (**e**) are shown in blue, NMR relaxation *S*^2^ in red and molecular dynamics simulation *S*^2^ in orange. Further comparisons are shown in Supplementary Fig. 3 the Supporting Information. **f** C1 and eNOE bundles with atoms 9H_2’_ and 10H_8_ colored in white, and the distances between them shown by red lines

Given the demonstrated high density of the eNOE network, we investigated if it is sufficient to calculate an ensemble of conformers where all states on average fulfill the distance restraints rather than each structure. We selected the 20 conformers with the lowest CYANA target function (TF) to represent the multi-state bundle. The TF for the 14-mer tetraloop RNA decreased with increasing number of states, indicating that multiple states were necessary to describe the input data (Fig. 4a). The largest decrease in the TF was from one to two states, which proceeded to level off from three to five states. Therefore, we chose two states to represent the multi-state bundle to avoid over-fitting the data. For many residues, it is possible to identify and group the two states, an example of which is shown (Fig. 4b). The two states are a result of the averaged nature of eNOEs, which causes the distance restraints between the guanine H_8_ proton and nearby protons to be shorter than the calculated single-state structure distances. Although the single-state structure places G10 H_8_ in a position that causes the least violation for all of the involved atoms, two states allow the eNOEs to be satisfied on average, capturing the local dynamics of the involved atoms in the structural ensemble. The overall heavy-atom RMSD of the two states is 0.84 Å, which is considerably larger than the 0.44 Å calculated for the single-state bundle. To assess the over-determination of the information content inherent to the eNOE-derived distance network, we performed a jack-knife analysis^54^. We prepared 10 sets of distance restraint files, where 10% of the restraints were randomly deleted, but each restraint only in one set. We calculated 10 ensembles with the remaining 90% of the input data, and determined partial target functions from the 10% omitted restraints in each structure calculation. Adding the 10 partial target function values results in a cross-validation target function in which the entire input data is represented. For the single-state bundle, this value was 40.6 Å^2^, and it dropped to 37.6 Å^2^ for the two-state ensemble. As we do not observe a further decrease for higher-state ensembles, we conclude that a two-state representation of the tetraloop is appropriate.

For a comparison of the spatial sampling represented by the two-state ensemble to a previous molecular dynamics simulation and to dynamics probed by previously published NMR relaxation data^55–57^, we calculated order parameters *S*^2^ for all N-H and C-H covalent bonds and P-OP_1_ from the two-state ensemble. *S*^2^ is 1 if the bond orientation is completely restricted and 0 if the bond has complete angular freedom. *S*^2^ values for C_5_/H_5_, C_6_/H_6_, C_8_/H_8_ and C_1’_/H_1’_ are shown (Fig. 4c, all other values in Supplementary Fig. 3 in the Supporting Information). The agreement for all base *S*^2^ is exceptionally good. For the sugar *S*^2^, the 1′ and 2′ positions are also excellent, whereas 4′ is moderate and 5′ and 5′′ are relatively poor. We note that the density of eNOEs involving 5′/5′′ was lower than for the other positions, which may explain the lower agreement. We also assessed the backbone dynamics be comparing ensemble P-O *S*^2^ to *S*^2^ derived from ^31^P CSA values^57^. Not surprisingly, the agreement is poor, as we do not have any restraints for P and O atoms. The comparison may be further challenged by the difficulty in disentangling the order parameter from the apparent CSA tensor. We note that there is also considerable disagreement between these relaxation *S*^2^ and a recent MD simulation of the tetraloop^58^. Overall, the two-state ensemble reproduces experimental order parameters well. We regard this as an independent confirmation of the representation of the spatial sampling of our two-state ensemble.

## Discussion

In conventional structure calculation of RNA, many ad hoc restraints such as hydrogen bond or planarity restraints are used and combined with labor-intensive additional experiments. Here, we have demonstrated that we can reproduce high-resolution structures of small RNA based on eNOE restraints alone. Conventional semiquantitative NOE upper distance bounds discard a particularly large part of structural information regarding medium- to long-range features and dihedral angles that is actually encoded in the cross-relaxation rate^52, 53^. However, the eNOEs are able to accurately determine this kind of information for RNA. For instance, the stem loop curvatures defined by the conventional NOEs and eNOEs are quite different, suggesting that the eNOEs are able to pick up on the relative orientation of the stem loop (Fig. 3c). This is further illustrated by comparing the backbones of the eNOE structure (Fig. 3h, black), 2KOC (Fig. 3h, cyan), and the conventional NOE structure (Fig. 3h, red), which shows that the eNOE structure aligns much better with 2KOC than to the structure from conventional NOEs. This demonstrates that eNOEs are indeed capable of defining the orientation of the stem relative to the loop. This is a cumulative effect resulting from many accurate short-range restraints rather than direct long-range restraints. It is worth to mention that the curvature of the bundle calculated from conventional NOEs does not improve upon inclusion of angular restraints that restrict torsion angles to their generally allowed regions as proposed in reference^59^.

Although ad hoc restraints are very helpful in defining average structures, they cannot be used to assess spatial sampling. They may even produce erroneous sampling because no detailed relationship between indirect evidence and parametrization of specific conformations in a dynamic ensemble is possible. We have previously shown that in structure calculations that try to fulfill all restraints with a single structural state, the accuracy of our eNOEs results in high TF values indicative of many distance restraint violations^24^. This results from the eNOE’s ability to pick up on structural dynamics, which is normally suppressed in a single-state structure calculation as the algorithm attempts to finds the global minimum that simultaneously satisfies the distant restraints best. Thus, allowing for multiple states in the structure calculation alleviates these disagreements and allows for multi-state structures to be calculated that sample their conformational space^24, 53^.

The two states obtained for the cUUCGg tetraloop are marked by a clear difference in the relative positioning of the backbone, as well as differences in the location of the bases with respect to each other. The nucleobases of U6 and G9, for example, preserve Watson–Crick base pairing as they undergo correlated motions between the two states. A similar trend is present for the C5 to G10 base-pair, although this particular base-pair appears to sample a larger angular space than that of U6 to G9. The positioning of the U6 to G9 base-pair in the two states also appears to influence the orientation of the C8 base relative to U6. Interestingly, U6 in the first state is further away from the loop than in the second state, which would mean that base-stacking between U6 and C8 would be less favorable than in the second state where U6 is located closer to the loop. The backbone is also correlated to these changes. It is noteworthy that the distinction between the two states in the loop is progressively lost down the stem, suggesting that the stem and the loop undergo motions that are not correlated. Although the motions sampled by the eNOEs are of little biological significance for the thermostable UUCG tetraloop, they may be of interest for investigations of RNA systems where dynamics play a critical role in the modulation of their functions.

There were some rather large changes between distances calculated from the C1 ensemble by *r*^−6^ averaging, which takes into account motional effects, and the ensemble linearly averaged distances < *r* > . To demonstrate how eNOEs are sensitive to dynamics, we investigated one of the largest outliers (Fig. 4f). Here, the two methods of distance calculation yield more than a 0.5 Å difference for the 9H_2′_–10H_8_ distance from C1 (3.93 Å from *r*^−6^ averaging and 4.50 Å by arithmetic average). The 2KOC bundle is extremely tight and thus showed almost no difference between the two methods. The difference between the two methods of distance calculation for C1 is due to a rather large amount of rotational dynamics of the bases G9 and G10 (Fig. 4b). This base twisting causes atoms 9H_2’_ and 10H_8_ to undergo large fluctuations relative to each other (see Fig. 4f), which in turn augments the measured *σ* and thus decreases the extracted effective eNOE distance. In line with this, the extracted eNOE distance between this atom pair of 3.97 Å (Supplementary Data File 1) was extremely close to the *r*^−6^ calculated distance from C1 of 3.93 Å (Table 1). In addition, our two-state eNOE structure sampled a similar conformation space to that of C1 (Fig. 4f), although the linearly averaged distances from the two-state eNOE bundle resulted in a distance that was slightly shorter than the linearly averaged distance from C1.

**Table 1:** Distances extracted from either r^−6^ averaging or the ensemble linearly averaged distances 1/ < r > ^6^ from 2KOC, C1, or the eNOE two-state ensemble

2koc.pdb distance (Å)	3.94
C1 distance (Å) < 1/*r*^6^>	3.93
C1 distance (Å) < 1/ < *r* > ^6^	4.50
Two-state eNOE structure distance (Å) < 1/*r*^6^>	3.98
Two-state eNOE structure distance (Å) 1/ < *r* > ^6^	4.09

**Fig. 5:**
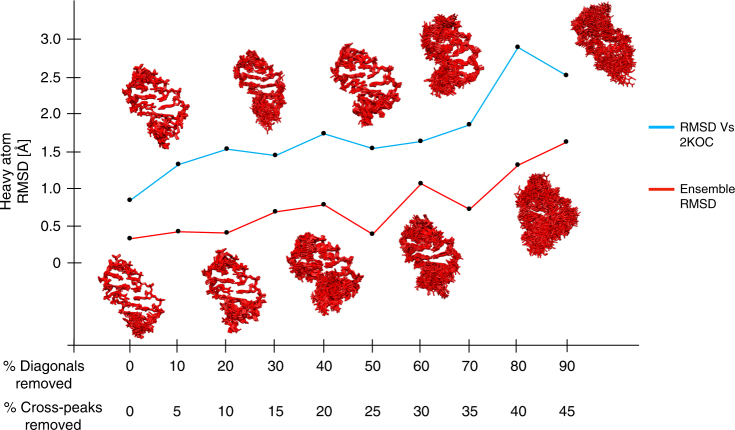
Simulation of eNOE analysis of larger RNA molecules. Trends of the RMSD value of the single-state bundle (red) and the RMSD from the previously determinated NMR structure 2KOC (blue) with increasing peak overlap are shown. eNOE restraints were recalculated assuming that the percentage of original diagonal peaks that cannot be evaluated is twice as large as the corresponding percentage of the cross peaks, as shown on the *x* axis. The resulting bundles are shown for all calculations

The information density obtained from eNOEs is higher than the one contained in a conventional NOE network. As such, eNOEs should improve the structure calculation of RNAs of any size. A particularly interesting question is what quality of structures of RNA larger than the 14-mer studied here can be expected. The relevant parameter is the eNOE density, which is the number of diagonal and cross peaks that can be evaluated per nucleotide. Therefore, we simulated increasingly larger RNA constructs be deleting fractions of diagonal and cross peaks in a 2:1 ratio (Supplementary Fig. 4 in the Supporting Information and Fig. 5). The chosen ratio reflects the fact that the diagonal overlap increases faster than the cross-peak overlap, because only one resonance has to be similar for two spins. Bundles obtained from structure calculations with the new distance restraints are plotted along with the RMSD values of the bundles and the RMS deviation from the reference NMR structure 2KOC. There is an approximately linear increase in both the bundle RMSD and the deviation from 2KOC up to a loss of 70% diagonal peaks and 35% cross peaks, after which the structures deteriorate considerably (RMSD larger than 1 Å, RMS deviation from 2KOC larger than 2.5 Å). Beyond deleting 40/20% there is also a dependence on the exact selection of diagonal peaks to be deleted as indicated by the deviation of the general trend in the bundle RMSD. The reason for this observation is that the deletion of a specific diagonal peak shifts all distance restraints involving the corresponding atom into a less stringent category (bi-directional →  uni-directional →  generic normalized NOE), which presumably causes low eNOE density in specific segments. Rather than defining a specific size cutoff, we recommend estimating the number of diagonal and cross peaks that can be evaluated for a specific RNA under study, which can be compared with the plot presented in Fig. 5.

In conclusion, we have applied our eNOE protocol to a nucleic acid for the first time. We have established an estimation for the ideal maximal NOESY mixing time for eNOE measurements with RNA, and showed that eNOEs extracted from buildup curves are of high quality. We expect that eNOE data sets of similar quality can be obtained for larger RNA constructs employing ^15^N and ^13^C labeling. This strategy would also allow to measure more eNOEs between the ribose H_3′_-H_5′′_ protons, which should substantially improve the γ dihedral angles. We calculated a single-state structure of the 14-mer UUCG tetraloop to 0.44 Å precision with only eNOEs as input, which agreed well with the previously determined high-resolution structures that were calculated using conventional NOEs, RDCs, dihedral angles, planarity restraints, and Watson–Crick base pairing. We emphasize that eNOEs can be acquired in a fraction of the time compared with other restraints such as RDCs and dihedral angle restraints. In addition, the accuracy and precision of the eNOEs allowed us to calculate a two-state ensemble that samples its conformational space. For such multi-state calculations, only NOE and RDC restraints can be used, but not other popular semi-empirical ones. For these reasons, it is our hope that our eNOE protocol will be found useful among the NMR RNA community to help define RNA ensembles where a sufficient number of eNOE restraints can be collected. For larger RNA, where the NOE restraint density is not sufficient for the calculation of a structure, selective distances of high interest may be determined. We have recently demonstrated that distances between valine, leucine and isoleucine methyl groups can be measured in a 360 kDa protein complex^60^. With the implementation of eNORA2 into the newest version of CYANA, the computational procedure is straightforward to apply.

## Methods

### Sample conditions

Two 2.0 µmol scale synthesis reactions of unlabeled 14-mer-cUUCGg tetraloop RNA with the sequence 5′-PO_4_^2—^PO_3_^—^PO_2_-GGCAC(UUCG)GUGCC-3′ were purchased from Dharmacon (Lafayette, Colorado, United States) with High-Performance Liquid Chromatography (HPLC) purification. Both samples were re-suspended and then dialyzed via centrifugation in their respective buffers to remove residual triethylammonium from the synthesis. The first sample was concentrated to a final concentration of 1 mM and a total volume of 500 µl in 20 mM potassium phosphate, pH 6.4, 0.4 mM EDTA and 5% D_2_O. The second sample was concentrated to a final concentration of 1.7 mM and a total volume of 500 µl in 20 mM potassium phosphate, pH 6.4, 0.4 mM EDTA and 100% D_2_O.

### NMR data and processing

All spectra were acquired at 25 °C on a 900 MHz Direct Drive Varian spectrometer equipped with a 5-mm triple resonance ^1^H/^13^C/^15^N cryo-probe with *z* axis gradient. For the sample in H_2_O, a 2D WaterGate [^1^H-^1^H] NOESY buildup series with eight mixing points (40, 80, 120, 160, 200, 240, 280, and 320 ms) was acquired with 1470 × 200 complex points and a 1.3-s recycle delay. The number of scans was 64 and *t*_max_ was 73.5 ms in the direct and 10 ms in the indirect dimension. For the sample in 100% D_2_O, a 2D PreSat [^1^H-^1^H] NOESY buildup series with eight mixing points (40, 80, 120, 160, 200, 240, 280, and 320 ms) was acquired with 1470 × 400 complex points and a 1.5-s recycle delay. The number of scans taken was 32, and the *t*_max_ was again 73.5 ms in the direct dimension, and 20 ms in the indirect dimension. All spectra were processed with the NmrPipe/NmrDraw/NlinLS package^61^. Each dimension was apodized using a 90^o^ phase-shifted cosine-bell window function and zero-filled once. Assignment of peaks in the 320 ms NOESY spectra from both the H_2_O and D_2_O samples was done in ccpNMR^62^ based on the resonance assignments of the previously solved high-resolution structure^21^ (PDB entry 2KOC, BMRB entry 5705). Cross and diagonal peak intensities at all eight mixing times were extracted using the NlinLS autofit script with the assignment from the longest mixing time (320 ms) as the reference spectrum.

### NOESY buildup fitting and distance restraints using CYANA

Cross-relaxation rate constants (*σ*) and auto-relaxation rate constants (*ρ*) were determined using the full-matrix approach^14, 63^, which is a part of the eNORA2 package^64^ that has recently been implemented into the CYANA software package^65, 66^. The protein-specific MATLAB code of eNORA2 has been transcribed into the Fortran programing language and generalized for use with DNA/RNA and any other molecules that can be handled by CYANA^67^. The implementation in CYANA also extends features such as the three-spin approximation of spin diffusion for partially deuterated molecules to deuteration of any configuration. The increase in computational efficiency in the CYANA framework furthermore allows for spin diffusion averaging over several conformers. Details of the CYANA implementation of eNORA2 will be described elsewhere. The full-matrix approach corrects for spin diffusion by simulating the active magnetization transfer pathways between all spins simultaneously. Spin diffusion corrections were calculated from the existing NMR structure (PDB entry 2KOC)^21^, as well as from relevant atoms in a 10-state bundle provided by Hashim Al-Hashimi^15^ (C1). The spin diffusion corrections and the extracted eNOE distances depend on the overall rotational correlation time *τ*_*c*_ via the spectral density function^26^. This requires an accurate overall *τ*_*c*_ value as input. For the sample in H_2_O, we used the previously determined *τ*_*c*_ of 2.23 ns determined by NMR and molecular dynamics simulations^55^. For the sample in D_2_O, we used *τ*_*c* = _2.74 ns, which was estimated from the 23% viscosity increase of D_2_O using the Stokes–Einstein equation^68^. An average auto-relaxation rate constant *ρ* of 2.9 s^-1^ was used for spins for which no value could be fitted. The spin diffusion corrections at each mixing time were derived from the simulation and applied to the extracted intensities. The diagonal peaks were fit to monoexponential decay curves to determine *ρ*_*i*_ and *ρ*_*j*_ and initial magnetization values Δ*M*_*ii*_(0) and Δ*M*_*jj*_(0)^63^. The corrected cross-peak buildup curves were then fitted using *ρ*_*i*_, *ρ*_*j*_, Δ*M*_*ii*_(0), and Δ*M*_*jj*_(0) as fixed input parameters, and the cross-relaxation rate constants *σ*_*ij*_ and *σ*_*ji*_ as free variables. Uni-directional buildups that were of sub-par quality when normalized to the spin of origin (*i→j*), but were of high quality when normalized to the destination spin (*j→i*), were normalized to the destination spin^13^. The quality of all fits was evaluated visually, and poor fits from both (*i→j*) and (*j→i*) were excluded. Then, *σ*_*ij*_ and *σ*_*ji*_ were converted into distance restraints *r* through the relationship *σ* ~ *r*^−6^. Δ*M*_*ii*_(0), Δ*M*_*jj*_(0), *ρ*_*i*_, *ρ*_*j*_, *σ*_*ij*_, *σ*_*ji*_, and *r*’s were determined using the intensities from all eight mixing times (40–320 ms), as well as from the first four (40–160 ms). Further analysis described in the Supporting Information revealed that rates obtained from fits to 160 ms are more reliable and were used for structure calculation. For the extraction of the distances, we assumed isotropic tumbling of the molecule. Using the simulations (Fig. 3 of reference Vögeli et al.^22^), we estimate a maximal distance error of ca. 2% for a molecule with a ratio of 1.5 between the longitudinal and transverse axes of the diffusion tensor. In CYANA, this entire process is automated except a visual fit evaluation. Comparison of the extracted eNOE cross-relaxation rates *σ* from non-exchangeable resonances between the H_2_O and D_2_O NOESY buildup series showed a slope of 1.21, indicating that our *τ*_*c*_ approximation for the D_2_O sample was in good agreement with the data (Supplementary Fig. 5).

### Determination of ideal maximum mixing times

The proton distribution and density in RNA is different from that of proteins. Thus, we expect an optimal maximum mixing time that is different from the previously established tumbling time-dependent value for proteins^26^. Although most of the fits were similar in quality to those shown (Fig. 2c), there were some notable exceptions which required additional investigation. First, the diagonal peak intensities of ribose methylene protons, as well as the amino protons of guanine, adenine, and cytosine bases followed a similar pattern where the intensities apparently decayed much faster within the first four points than the last eight. Hence, the *ρ* and corresponding Δ*M*(0) (for amino protons only) values fitted from mixing times 40–320 ms were smaller than those fitted from 40–160 ms (Supplementary Fig. 6a and 1b, Supplementary Fig. 6d). This effect is caused by a deviation from monoexponential decay. We simulated decay curves for amino, methylene and all other atoms assuming typical *ρ* values and upper limits for effective *σ* values, which integrate the dipolar interactions with all protons (Supplementary Figs. 6f ). The true *ΔM*(0) values are underestimated by 12, 5, and 2% when fitting a monoexponential function to 40–160 ms. The effect is stronger for methylene or amino protons because their effective *σ* is dominated by the dipolar interaction with the geminal proton. These errors translate into relatively small distance errors of <2% even in the case of amino protons. However, fitting to 40–320 ms results in 45, 21, and 12% underestimation of *ΔM*(0) for the same extreme cases. The resulting distance errors of up to 6% (for amino protons) suggest that they can be reduced by restricting the fits of the diagonal peak decays to a maximal mixing time of 160 ms.

We also investigated the effect of fitting *σ* from 40 to 320 ms and from 40 to 160 ms mixing time on the rates, as well as the extracted distances. Correlation plots between *σ* from fitting from 40 to 320 ms and 40 to 160 ms indicated that the most extreme difference occurred for interactions within methylene and amino spin pairs (Supplementary Fig. 6e). Interestingly, *σ* from interactions between a single amino/methylene proton and a non-amino/non-methylene proton, or to an amino/methylene proton on a separate residue had similar values between fitting four and eight points (Supplementary Fig. 6e). However, it was clear that the eNOE distances from both scenarios were extremely close, as shown in Supplementary Fig. 6c. In addition, comparison of effective eNOE distances from fits to 160 ms and 320 ms vs the 2KOC and C1 structures showed very similar statistics (Supplementary Fig. 7a-f). Therefore, we decided to fit *ρ* and *σ* from 40 to 160 ms, as the corrections for spin diffusion, and thus the error, increase at longer mixing times. We regard this result as a general guideline for the optimal choice of the mixing time. Assuming an inverse relationship of the maximal mixing time with the overall tumbling time *τ*_*c*_, we obtain a maximal mixing time of 4 × 10^−10^ s^2^ *τ*_*c*_^-1^.

### eNOE distance comparison against 2KOC and C1

The extracted eNOE distances (with spin diffusion correction from 2KOC) from buildups of exchangeable resonances in H_2_O were combined with the distances from buildups in D_2_O to create two master lists with distances from fits to data from 40 to 320 ms and 40 to 160 ms mixing times, respectively. The distances from 40 to 160 ms are listed (Supplementary Data 1). The same process was repeated for distances calculated with spin diffusion corrections based on the relevant atoms of the C1 bundle. The eNOE distances determined with spin diffusion corrections from 2KOC or C1 were then compared with distances calculated from the 20-conformer 2KOC structure or C1 respectively by taking the *r*^−6^ average ( < 1*/r*^6^ > ), where *r* is the distance between two atoms and < > denotes the ensemble average. eNOE distances were also compared with the average distances < *r* > calculated from just C1.

### Structure calculations

Distances extracted from bi-directional eNOEs (both symmetry-related cross peaks can be normalized to their corresponding diagonals) had no error tolerance applied and had the same values for the upper and lower limit distance restraints. For uni-directional eNOEs (only one cross-peak can be evaluated or the eNOE cannot be normalized to both diagonals), a tolerance of ± 10% was applied for the conversion to upper and lower distance limits^13^. Generic normalized eNOEs (gn-eNOEs)^51^ were converted into upper distance limit restraints and given a tolerance of ± 10% (gn-eNOEs and corresponding distances are listed in Supplementary Data File, Table 2). gn-eNOEs were calculated by giving overlapped diagonals an upper limit Δ*M*(0) and *ρ*. Amino, methylene, and all other hydrogen atoms had substantially different Δ*M*(0) and *ρ* values, and therefore the upper limit Δ*M*(0) and *ρ* values were based on the highest values in the corresponding atom groups. All structure calculations were performed in CYANA-3.98^65, 66^, starting with 100 initial structures with random torsion angle values using the standard simulated annealing protocol with 10,000 torsion angle dynamics steps. The 20 structures with the lowest target function values were selected for the ensemble. For the structure calculation based on conventional NOEs, a total of 677 upper distance limit restraints were used as input. For the single-state structure calculated from eNOEs, a total of 75 bi-directional eNOEs, 190 uni-directional eNOEs, and 88 gn-eNOEs were used. The multi-state structures were calculated as previously described^24^ using the same input restraints as for the single-state structure. The symmetry restraint weight was 0.1 for all heavy atoms with a flat-bottom width of 1.2 Å in a harmonic potential.

### Data availability

The final structure coordinates and processed spectra were deposited into the PDB/BMRB database (BMRB ID 30386; PDB IDs 6BY4 and 6BY5, respectively, for the single- and two-state ensemble).

## Electronic supplementary material

Supplementary Information

Description of Additional Supplementary Files

Supplementary Data 1
