# Supplementary material for: High-resolution small RNA structures from exact nuclear Overhauser enhancement measurements without additional restraints
Source: Commun Biol. 2018 Jun 7;1:61. doi: 10.1038/s42003-018-0067-x (PMC6123705; doi:10.1038/s42003-018-0067-x)
Supplement: Supplementary file 1 — Supplementary Information [file 42003_2018_67_MOESM1_ESM.pdf]

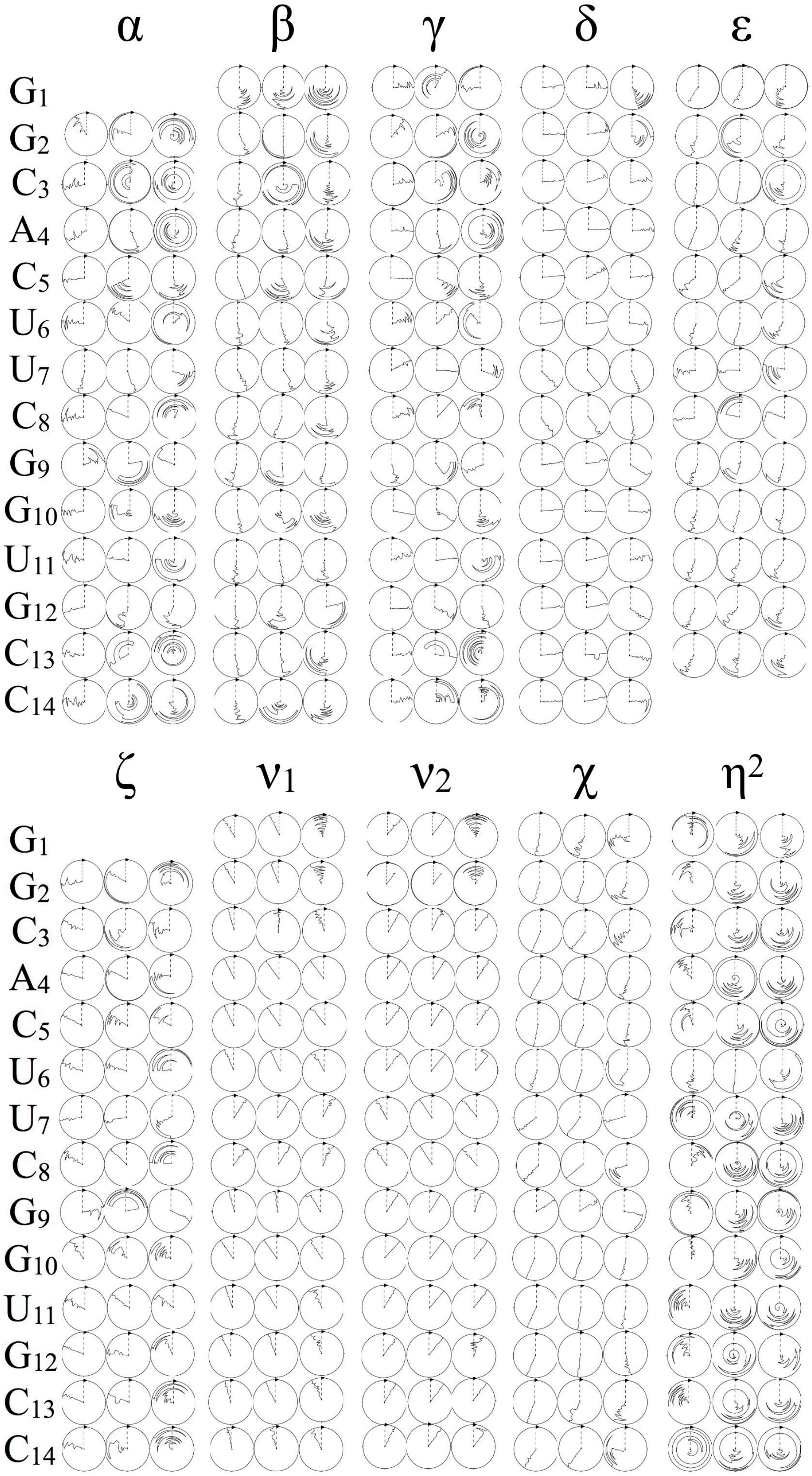
α
β
γ
δ
ε
G_1
G_2
C_3
A_4
C_5
U_6
U_7
C_8
G_9
G_{10}
U_{11}
G_{12}
C_{13}
C_{14}
ζ
ν_1
ν_2
χ
η^2
G_1
G_2
C_3
A_4
C_5
U_6
U_7
C_8
G_9
G_{10}
U_{11}
G_{12}
C_{13}
C_{14}

Supplementary Figure 1. Improved definition of eNOE-based dihedral angles.
Circle plots of dihedral angles α, β, γ, δ, ε, ζ, ν_1, ν_2, χ, and η_2, were calculated using MOLMOL[54]. The angles of each member of the ensembles are plotted starting from the center towards the outer circle. The full circle covers a range of 0 (indicated by the arrow on top) to 360 degrees in clockwise rotation. The three circle plots for each dihedral angle refer to 2KOC (left), the eNOE structure (middle), and the conventional NOE structure (right), respectively.

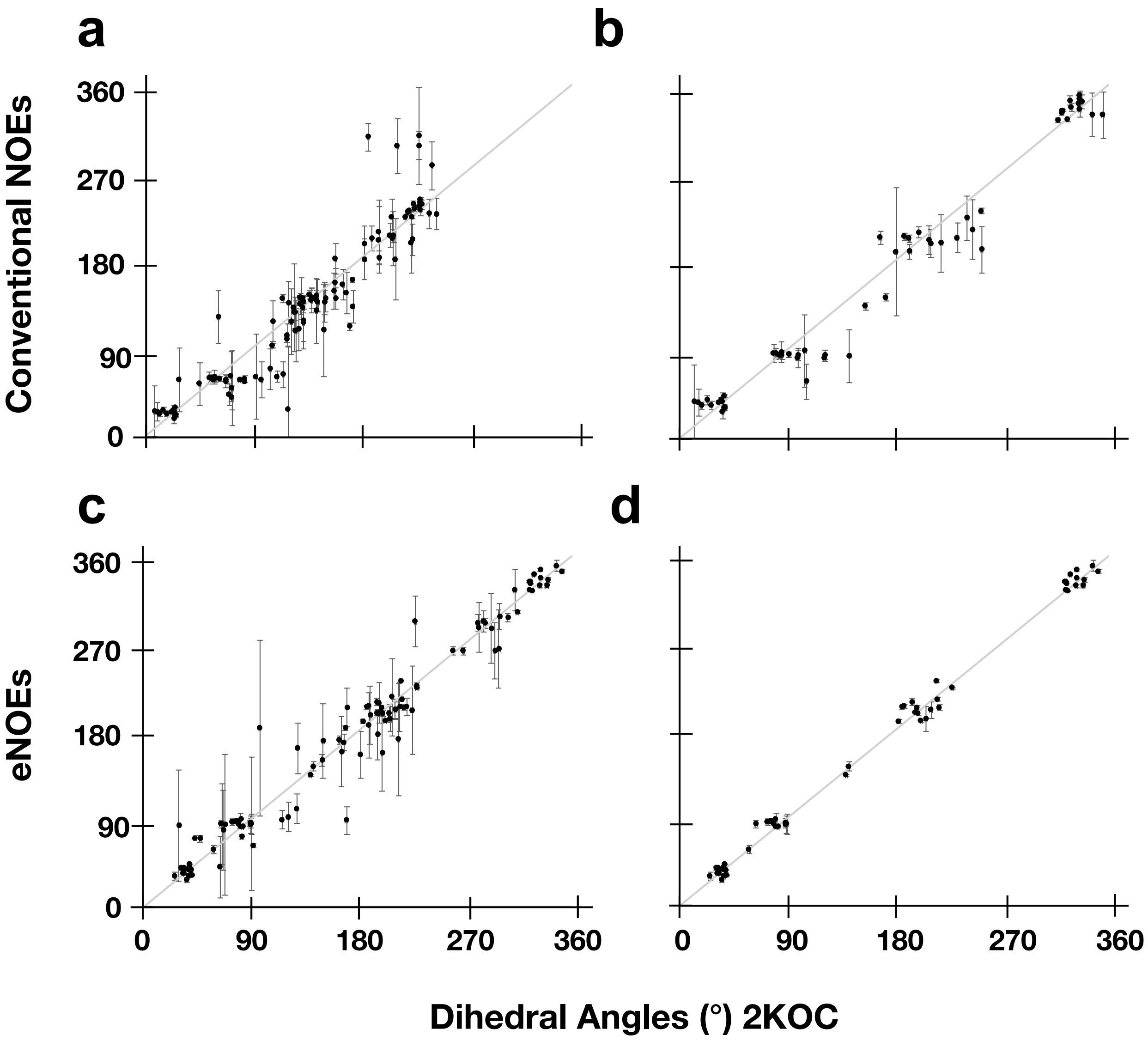

Supplementary Figure 2. Comparison of dihedral angles from eNOEs and conventional NOEs.

a. Correlation plot between β, γ, δ, ε, ζ, χ, ν_1, HOXI from U6, and ν_2 dihedral angles from 2KOC on the *x*-axis and the NOE structure on the *y*-axis ($y = 0.99x$, $R = 0.88$).

b. Correlation plot between δ, χ, ν_1, HOXI from U6, and ν_2 dihedral angles from 2KOC on the *x*-axis and the NOE structure on the *y*-axis ($y = 0.98x$, $R = 0.98$).

c. The same correlation plot as in **a**, except with dihedral angles from the eNOE structure on the *y*-axis ($y = 0.99x$, $R = 0.98$).

d. Same as in **b**, except with angles from the eNOE structure ($y = 1.00x$, $R = 1.00$).

For all correlation plots, negative angles were converted into positive ones by the addition of 360°. An addition of 360° was also added to positive angles when it minimized the difference between 2KOC and the NOE or eNOE structure. The error bars indicate the standard deviation of the angles within a specific structural bundle.

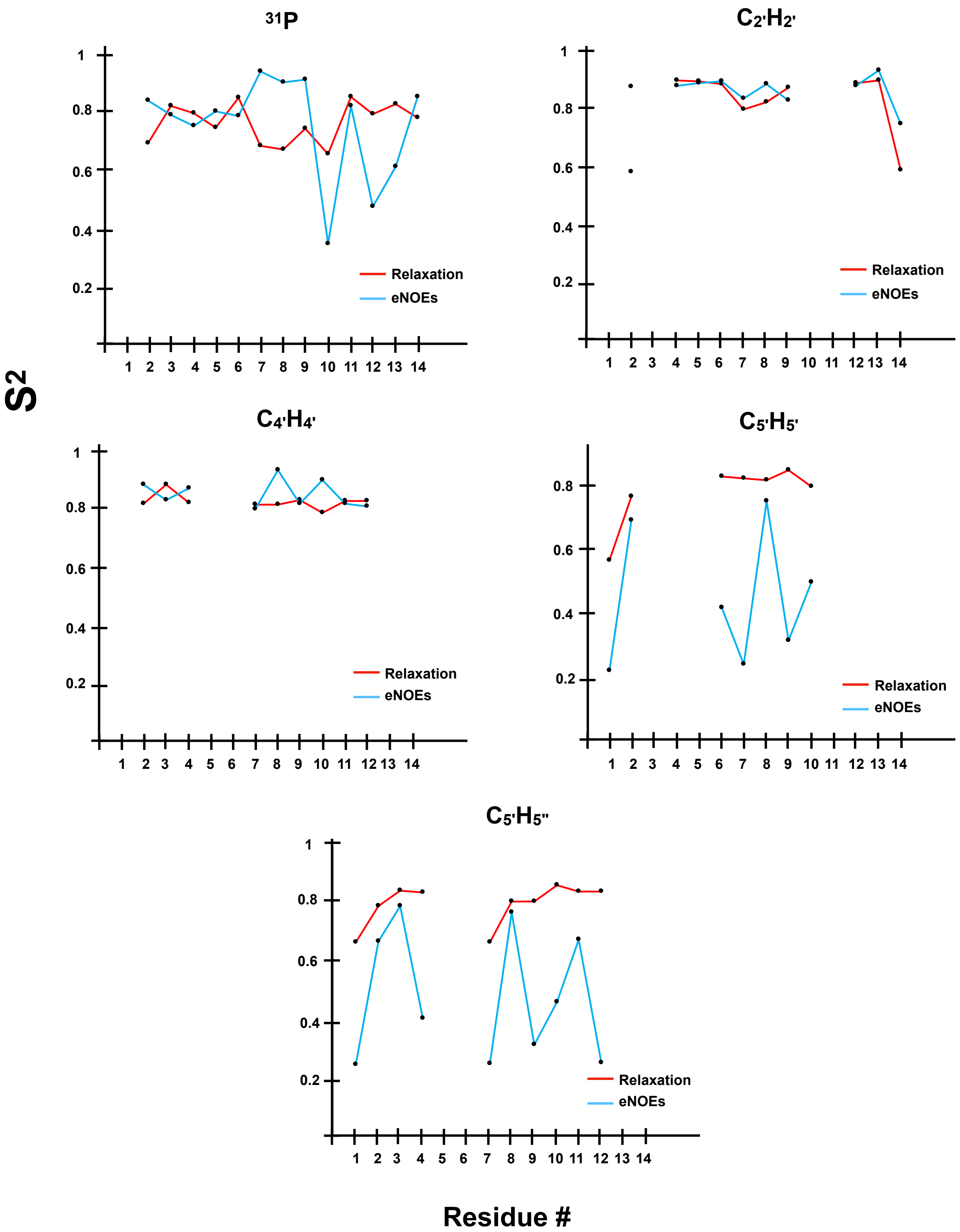

Supplementary Figure 3. Back-calculated order parameters S^2 from the two-state eNOE ensemble. Back-calculated S^2 values from the two-state eNOE ensemble for the atoms shown are plotted in blue. ^{31}P S^2 values were obtained from [59], and are shown in red. $C_{2'}$-$H_{2'}$, $C_{4'}$-$H_{4'}$, $C_{5'}$-$H_{5'}$, and $C_{5'}$-$H_{5''}$ S^2 values were obtained from [58], and are also shown in red.

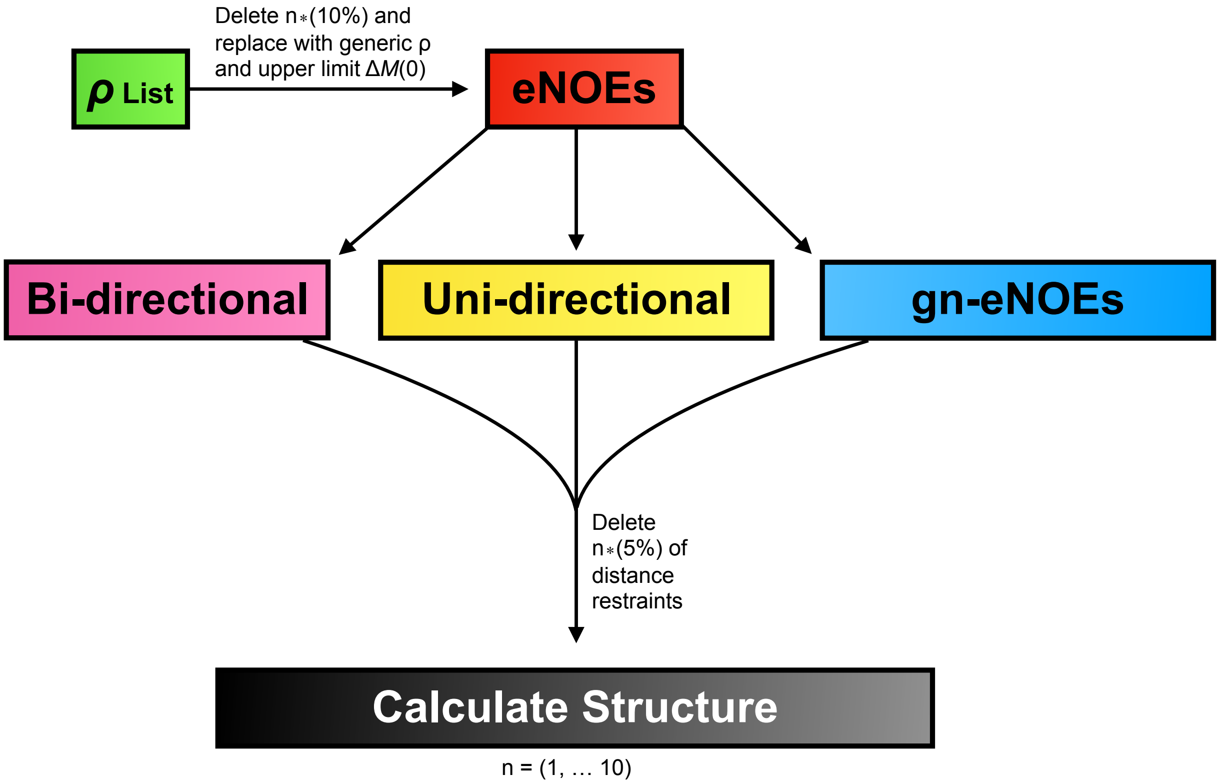

Supplementary Figure 4. Strategy for the simulation of larger RNAs.
First, *n* (where *n* is an integer from one to 9) multiplied by 10% of the original diagonal peaks are deleted. Second, *n* multiplied by 5% of the resulting bi-directional and uni-directional eNOEs, as well as an additional 5% of the gn-eNOEs, were deleted to simulate cross-peak overlap, and a structure calculation was performed in CYANA with the remaining restraints.

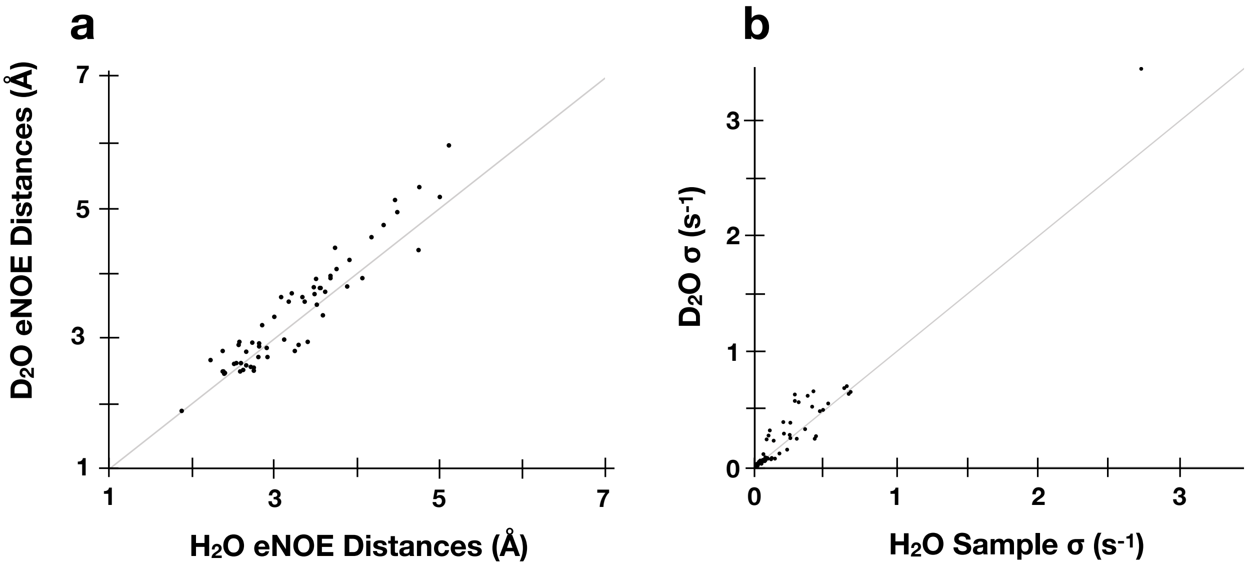

Supplementary Figure 5. Correlation plot between distances and σ values extracted from H_2O and D_2O buildups.

A correlation plot between the distances extracted from eNOEs between non-exchangeable resonances in H_2O on the x-axis and D_2O on the y-axis is shown in **a** ($y = 1.04x$, $R = 0.95$). In **b**, a correlation plot between the fitted σ of the corresponding distances in **a** are shown ($y = 1.21x$, $R = 0.97$).

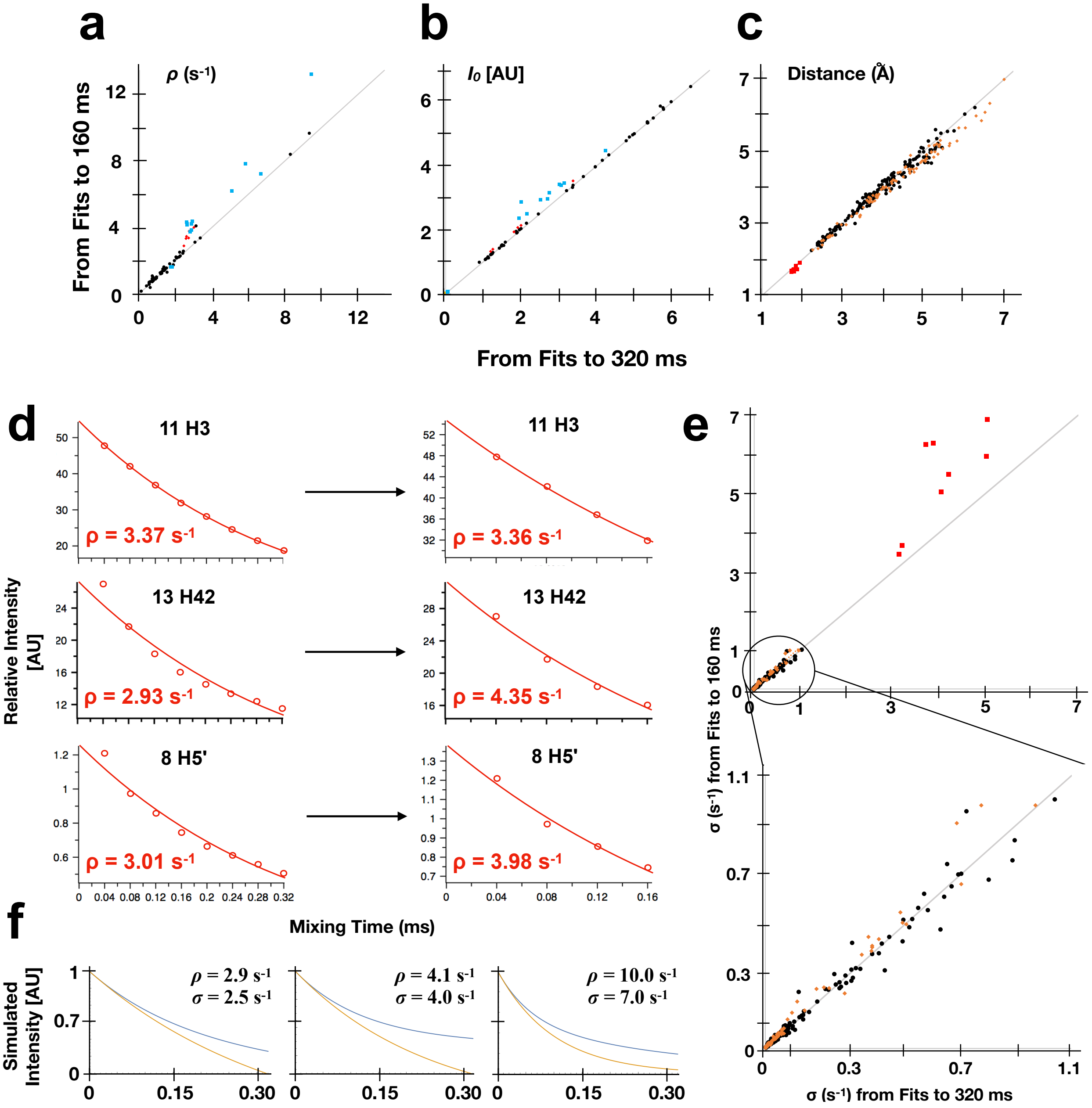

Supplementary Figure 6. Dependence of fits on maximum mixing times.

a. Correlation plot between ρ rates fitted from 40–320 ms on the x-axis versus 40–160 ms on the y-axis. Black circles are ρ values from non-amino/non-methylene protons ($y = 1.03x$, Pearson's correlation coefficient $R = 1.00$). Blue squares are ρ values from amino protons ($1.32x$, 0.97). Red diamonds are ρ values from methylene protons ($1.28x$, 0.88).

b. Correlation plot between $\Delta M(0)$ values extrapolated from fits from 40–320 ms on the *x*-axis versus 40–160 ms on the *y*-axis. Black circles ($y = 1.00x$, $R = 1.00$), blue squares ($1.13x$, 0.99), and red diamonds ($1.06x$, 1.00) correspond to non-amino/non-methylene, amino, and methylene protons respectively.

c. Correlation plot between eNOE distances from fitting from 40–320 ms on the *x*-axis versus 40–160 ms on the *y*-axis (overall correlation: $y = 0.98x$, $R = 0.99$). Black circles are eNOE distances between non-methylene/non-amino protons ($0.99x$, 0.99), orange diamonds between amino/methylene and non-amino/non-methylene or amino/methylene protons on a different residue ($0.96x$, 0.99), and red squares within methylene/amino spin pairs ($0.96x$, 0.85).

d. Mono-exponential decay curves for $11H_3$ (top), $13H_{42}$ (middle), and $8H_{5'}$ (bottom) from fitting to 40–320 ms (left) vs 40–160 ms (right).

e. Correlation plot between the fitted σ rates from 40–320 ms on the *x*-axis and 40–160 ms on the *y*-axis. The black dots are σ between non-amino/non-methylene protons ($y = 0.97x$, $R = 0.99$). The orange dots are σ between an amino/methylene proton and a non-amino/non-methylene proton or an amino/methylene proton on another residue ($1.09x$, 0.99). The red dots are σ within amino/methylene spin pairs ($1.33x$, 0.76). The bottom figure shows the same correlation without the series from σ within amino/methylene spin pairs ($1.01x$, 0.99). The overall correlation for all series is ($1.30x$, 0.99).

f. Deviation from monoexpontential behavior of diagonal peaks. Simulated diagonal decay curves for $\rho = 2.9\ s^{-1}$ and $\sigma = 2.5\ s^{-1}$ (left), $\rho = 4.1\ s^{-1}$ and $\sigma = 4.0\ s^{-1}$ (middle), $\rho = 10.0\ s^{-1}$ and $\sigma = 7.0\ s^{-1}$ (right) are shown in blue. For comparison, corresponding curves decaying monoexpontentially with the same ρ value are shown in orange. Monoexponential fits to the simulated decay curves up to 160 ms result in an underestimation of the initial magnetization of 2, 5 and 12%. The apparent ρ values are $2.3\ s^{-1}$, $2.6\ s^{-1}$ and $6.2\ s^{-1}$. For fits up to 320 ms, the initial magnetization is underestimated by 12, 21 and 45%. The apparent ρ values are $1.1\ s^{-1}$, $0.6\ s^{-1}$ and $0.9\ s^{-1}$.

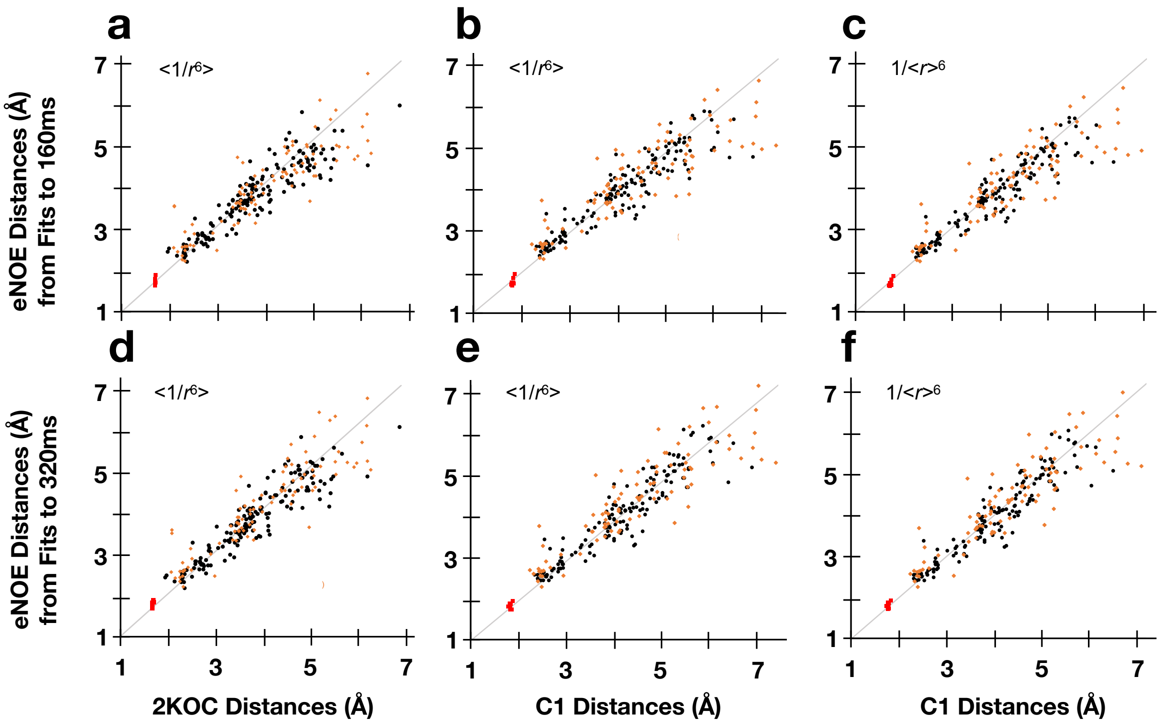

Supplementary Figure 7. Agreement between eNOEs and distances from 2KOC and C1.

a. Correlation plot between distances from 2KOC on the *x*-axis and eNOE distances from fits of 40–160 ms on the *y*-axis (overall correlation: $y = 0.96x$, $R = 0.89$). The black circles correspond to distances between non-amino/non-methylene protons ($y = 0.96x$, $R = 0.89$), the orange diamonds to distances between amino/methylene protons and non-amino/methylene or amino/methylene protons on a different residue ($y = 0.96x$, $R = 0.84$), and the red squares to distances within amino/methylene spin pairs (0.98*x*, 0.35). The 2KOC distances were determined by taking r^{-6} average (<1/r^6>).

b. Correlation between back-predicted distances from C1 on the *x*-axis and eNOE distances from fits of 40–160 ms (overall correlation: $y = 0.97x$, $R = 0.89$; black circles: 0.98*x*, 0.90, orange diamonds: 0.96*x*, 0.80, red squares: 0.98*x*, 0.60) on the *y*-axis. The back-predicted distances were calculated using the same method as in **a**.

c. The same correlation plot as **b**, except the eNOEs were compared to linearly averaged distances <r> from C1 (overall correlation: $y = 0.95x$, $R = 0.89$, black circles: 0.96*x*, 0.91, orange diamonds: 0.94*x*, 0.82, red squares: 0.98*x*, 0.59).

d. Correlation plot between back-predicted distances from the 20-conformer 2KOC bundle on the *x*-axis and eNOE distances determined by fits from 40–320 ms on the *y*-axis (overall correlation: $y = 0.98x$, $R = 0.91$). The black circles correspond to distances between non-methylene/non-amino protons (0.97*x*, 0.91), the orange diamonds to distances between an amino/methylene proton and a non-amino/non-methylene proton or a methylene/amino prpton on a different residue (0.99*x*, 0.87), and the red squares to distances within amino/methylene spin pairs (1.03*x*, 0.48). The distances from 2KOC were calculated by taking the r^{-6} average (<1/r^6>).

e. Correlation plot between back-predicted distances from C1 on the *x*-axis and eNOE distances determined by fits from 40–320 ms on the *y*-axis (overall correlation: $y = 1.00x$, $R = 0.91$). The individual colored series correspond to the same distance pairs as in **a** (black circles: 0.99*x*, 0.93, orange diamonds: 1.00*x*, 0.86, red squares: 1.02*x*, 0.39). The back-predicted distances were determined by the same method as in **a**.

f. Same plot as in **e**, except the distances from C1 were calculated by taking the linearly averaged distance <r> (overall correlation: $y = 0.98x$, $R = 0.92$, black circles: $0.98x$, 0.93), orange diamonds: $0.99x$, 0.86, red squares: $1.02x$, 0.40).
