# Supplementary material for: High-resolution small RNA structures from exact nuclear Overhauser enhancement measurements without additional restraints
Source: Commun Biol. 2018 Jun 7;1:61. doi: 10.1038/s42003-018-0067-x (PMC6123705; doi:10.1038/s42003-018-0067-x)
Supplement: Supplementary file 2 — Description of Additional Supplementary Files [file 42003_2018_67_MOESM2_ESM.docx]

**Description of Additional Supplementary Files**

File Name: Supplementary Data 1

Description: Excel worksheet containing a table with distances derived from bi- and uni-directional eNOEs, and a table with upper distance limits derived from generic normalized eNOEs.
